# Molecular Mechanisms Underpinning Immunometabolic Reprogramming: How the Wind Changes during Cancer Progression

**DOI:** 10.3390/genes14101953

**Published:** 2023-10-17

**Authors:** Irene Flati, Mauro Di Vito Nolfi, Francesca Dall’Aglio, Davide Vecchiotti, Daniela Verzella, Edoardo Alesse, Daria Capece, Francesca Zazzeroni

**Affiliations:** Department of Biotechnological and Applied Clinical Sciences (DISCAB), University of L’Aquila, Via Vetoio, Coppito 2, 67100 L’Aquila, Italy; irene.flati@graduate.univaq.it (I.F.); mauro.divitonolfi@univaq.it (M.D.V.N.); francesca.dallaglio@graduate.univaq.it (F.D.); davide.vecchiotti@univaq.it (D.V.); daniela.verzella@univaq.it (D.V.); edoardo.alesse@univaq.it (E.A.); francesca.zazzeroni@univaq.it (F.Z.)

**Keywords:** immunometabolism, cancer, immunosuppressive TME, NF-κB, PI3K/Akt/mTOR, AMPK

## Abstract

Metabolism and the immunological state are intimately intertwined, as defense responses are bioenergetically expensive. Metabolic homeostasis is a key requirement for the proper function of immune cell subsets, and the perturbation of the immune–metabolic balance is a recurrent event in many human diseases, including cancer, due to nutrient fluctuation, hypoxia and additional metabolic changes occurring in the tumor microenvironment (TME). Although much remains to be understood in the field of immunometabolism, here, we report the current knowledge on both physiological and cancer-associated metabolic profiles of immune cells, and the main molecular circuits involved in their regulation, highlighting similarities and differences, and emphasizing immune metabolic liabilities that could be exploited in cancer therapy to overcome immune resistance.

## 1. Introduction

Cancer is a metabolically dynamic disease, and metabolic reprogramming is one of the hallmarks of cancer [1,2]. Throughout disease progression, the metabolic needs of cells in the tumor microenvironment (TME) change, leading to a spectrum of metabolic phenotypes, where both glycolysis and mitochondrial respiration can support tumorigenesis in a cell- and context-specific manner [3]. Human tumor cells co-exist with immune and non-immune populations, such as stromal and endothelial cells. Altogether, these cell subsets constitute an integrated cancer model that characterize the tumor from a prognostic and therapeutic point of view [4]. Although the intimate connection between metabolism and the immunological state—the so called “immunometabolism”—is well established, little is known about the metabolic modulation of immune cells during cancer progression. At the early stages of tumorigenesis, immune cells try to antagonize cancer cells, that in turn attempt to escape immunological surveillance. TME could be infiltrated by tumor-associated tertiary lymphoid structures (TA-TLSs), mainly enriched in tumor-infiltrating lymphocytes (TILs), which are responsible for supporting antitumor immunity [5]. At advanced stages of tumorigenesis, the metabolic activity of cancer cells affects the function and the fitness of immune cells by contending for essential nutrients or producing waste metabolites that interfere with their activity. Hence, tumor-infiltrating immune cells are exposed to nutrient fluctuations and metabolic changes in the TME. Such alterations include variations in K^+^ ions, ATP, and adenosine concentrations [6]. Moreover, hypoxia and acidity caused by improper lactate accumulation contribute to create disadvantaged conditions for immune cells, thus impairing their physiological functions [7]. In this context, immunosuppressive populations, including tumor re-educated myeloid-derived suppressor cells (MDSCs), regulatory T cells (Treg), B cells, and tumor-associated macrophages (TAMs), promote immune escape, angiogenesis, and metastasis [8]. Hence, while on one hand, tumors regulate the availability of nutrients in the TME and cause the dysfunction of tumor-infiltrating anticancer immunity, on the other hand, immunosuppressive populations adapt to environmental conditions by modulating their metabolic pathway and fostering tumor progression.

In this review, we will describe the main metabolic circuits regulating immunometabolism across different immune subsets, and how these metabolic programs are rewired in cancer to favor immune escape. The comprehension of the molecular mechanisms underpinning immunometabolism and their deregulation during cancer progression represent a crucial step forward to exploit the therapeutical potential of metabolically reprogramming the tumor–immune cell interface, thereby improving the efficacy of existing immunotherapy. 

## 2. Signaling Pathways Regulating Immunometabolism

Several signaling pathways regulate catabolic and anabolic processes to maintain the immune homeostasis and functional specificity of immune cell subsets. Among these pathways, we can mention the Proliferator–Activated Receptor (PPAR) axis, and autophagy, but the most prominent role seems to be played by AMPK-LKB1, PI3K-Akt-mTOR, and NF-κB signaling that will be described below (Table 1).

*LKB1-AMPK signaling*. AMPK is acronymous of AMP-activated protein kinase. AMPK is composed of three subunits; AMPKα is the catalytic one, while AMPKβ and AMPKγ are the regulatory components. The activating phosphorylation on AMPKα can be mediated by several factors, including AMP/ADP-AMPKγ complex, LKB1, and the Ca^2+^/calmodulin (CaM)-dependent protein kinase 2 or β (CAMKK2 or β) [36]. Recently, Liu and colleagues identified that AMPK activation could be potentiated by the long noncoding RNA (lncRNA): NBR2. AMPK actives NBR2 which, in turn, sustains AMPK activity in response to prolonged stress stimuli, mainly during glucose starvation. AMPK acts as a metabolic sensor in a complex, including the liver kinase B1 (LKB1), a well-known tumor suppressor [37,38]. In particular, being sensible to intracellular ATP rate, AMPK is involved in switching on catabolic processes to restore consumed ATP content and switching off anabolic processes that sustain cell growth [39].

*PI3K-Akt-mTOR signaling*. PI3Ks are members of the Serine (Ser)/Threonine (Thr) kinase family that differ in their structure and substrate specificity. The PI3ks family is composed of classes I, II, and III, although class I is the most represented [40]. Class I members are divided into two subcategories, class IA and class IB enzymes, and both classes are activated in response to cell surface receptor stimulation. Receptor tyrosine kinases (RTKs), cytokine receptors, integrins, and G-protein-coupled receptors (GPCRs) are upstream inducers of class IA enzymes. GPCRs are the only receptors that also activate class IB enzymes [41,42]. Upon activation of PI3K at the plasma membrane, the phosphorylation of its substrate—phosphatidylinositol 4,5-bisphosphate (PIP2)—produces the second messenger phosphatidylinositol 3,4,5-trisphosphate (PIP3). Phosphatase and tensin homolog (PTEN) negatively regulates PI3K signaling by dephosphorylating PIP3 in PIP2 [41,42]. Akt or protein kinase b (PKB) is an effector of PI3K and is activated when its pleckstrin homology (PH) domain binds to PIP3. Upon stimulation, Akt undergoes conformational changes and initiates a signaling cascade involved in cell survival, growth, and proliferation [43]. The PI3k/Akt pathway induces glucose influx through TXNIP (thioredoxin-interacting protein) phosphorylation, which is necessary to reduce class I glucose transporters (GLUTs) endocytosis [44]. Moreover, the PI3k/Akt network regulates the activation of some glycolytic enzymes such as HK2 and LDHA [45,46]. The above-mentioned Akt functions support its involvement in promoting aerobic glycolysis. 

Current data also support the role of the PI3k/Akt pathway in lipid metabolism, where it regulates PPAR-α expression and SREBP1-mediated lipogenesis [47,48,49]. PI3K/Akt signaling also interacts with the pentose phosphate pathway (PPP). Indeed, PI3K/Akt breaks the interaction between glucose-6-phosphate dehydrogenase (G6PD), the rate-limiting enzyme of the PPP pathway, and its inhibitor TIRM21. The produced PPP metabolites consolidate Akt activation by establishing a positive feedback loop and downregulating the activity of the Akt inhibitor PHLDA3 [50]. Among the targets of Akt, glycogen synthase kinase 3 (GSK3), in the absence of Akt-mediated phosphorylation, is implicated in cellular metabolism by inhibiting its homonymous substrate glycogen synthase, thus blocking glycogen synthesis [51]. Moreover, GSK3 prevents de novo lipid synthesis by inducing the degradation of SREBP15. Other downstream effectors of Akt are the mammalian target of rapamycin (mTOR) and forkhead box O (FOXO) transcription factors. mTOR is a catalytic part of two multi-protein kinase complexes, mechanistic target of rapamycin complex 1 (mTORC1) and complex 2 (mTORC2). mTOR works as a sensor of nutrient levels and growth signals and regulates the balance of anabolic and catabolic metabolism. mTORC1 exploits available energy to stimulate anabolic processes, thereby sustaining the synthesis of macromolecules to promote cell growth and proliferation [52]. Instead, mTORC2 phosphorylates Akt and activates glycolysis, promoting GLUTs expression and hexokinase 2 (HK2) and phosphofructokinase-1 (PFK1) activation, and is also involved in lipid metabolism. Notably, mTOR and AMPK signaling constantly interact with each other to fine-tune cellular metabolism in response to environmental conditions [53].

*NF-κB signaling*. In mammals, the NF-κB transcription factor family includes five members named p65 (RELA), RELB, REL, NF-κB1 (p105/p50), and NF-κB2 (p/100/p52) [54]. All members form homo- or heterodimers, with p65/p50 being the most abundant one [54]. In the canonical pathway, NF-κB dimers are retained in the cytoplasm by IκB proteins. Upon activation by a variety of stimuli, including inflammatory cytokines, pathogen-associated molecular patterns (PAMPs) and molecules released by host cells and stress signals, the IκB kinase complex (IKK) phosphorylates IκB inhibitors, which, in turn, are polyubiquitinated and subsequently degraded by the 26S proteasome. Consequently, p65/p50 complexes are released and free to translocate to the nucleus. NF-κB is a stress sensor; therefore, it is not surprising that it plays a crucial role in governing metabolic adaptations to environmental changes and disruptions of tissue homeostasis. Indeed, NF-κB is activated in response to decreases in oxygen availability [55], glutamine and glucose fluctuations [56,57], and changes in energy provision through oxidative phosphorylation (OXPHOS), lipolytic pathway and other metabolic stimuli, both in normal and cancer cells [56,57,58,59]. Under hypoxic conditions, NF-κB promotes hypoxia-inducible factor-1α (HIF-1α) transcription. The following interaction between HIF-1α and HIF-1β allows for the activation of target genes containing hypoxia response elements (HRE) [60]. Recently, in mammalian cells and drosophila, a feedback mechanism was characterized, in which HIF-1α depletion causes NF-kB target gene increment via TAK–IKK complex [61]. These data suggest a key role of HIF-1α in limiting NF-κB pro-inflammation activity during inflammation and immune response [61]. Moreover, NF-kB regulates c-Myc expression, which, in turn, interacts with HIF-1α by establishing a very complex network, especially in cancer settings. Indeed, c-Myc activation has been associated with an increased mitochondrial biogenesis [62] and an increased rate of OXPHOS [62], fatty acid oxidation (FAO) [63,64], and glycolysis [65], making this transcription factor a master regulator of metabolism. 

## 3. Metabolic Life of Immune Cells

Due to their specific functions in the immune system, it is not surprising that each subset of immune cells presents a specific metabolic profile (Table 1). Environmental conditions and nutrient availability strictly regulate metabolic phenotypes determining cell proliferative capacity [66], and the switching between activation and quiescent states [67] in both resident and migrating cells. Depending on spatial localization, in fact, environmental stimuli, oxygen, pH, and metabolite availability influence cell metabolic profile that in turn modify nutrient sensing and migratory capacity, leading to a context-specific immune responses [68,69].

### 3.1. Macrophage Immunometabolism

Macrophages are phagocytic cells that reside in tissues or derive from circulating-monocyte differentiation. Macrophages are involved in innate immune response and are deputed to defend the organism against pathogens [70], remove apoptotic cells, and promote tissue regeneration and homeostasis [71]. In fact, macrophages respond dynamically to microenvironmental changes. For simplicity, macrophages are categorized into two different activation or “polarization” states: pro-inflammatory or classically activated macrophages (M1) and anti-inflammatory or alternatively activated macrophages (M2). Instead, the naïve M0 phenotype is the non-active form that can be stimulated by specific cytokines to differentiate toward a particular subset: GM-CSF promotes M1 macrophages while M-CSF induces M2 macrophage differentiation [72]. Each macrophagic phenotype is associated with a specific metabolism that supports polarization and functional demands [9]. 

M1 macrophage metabolism mainly relies on the activation of the glycolytic pathway. Accordingly, the inhibition of glycolysis impairs phagocytosis, redox status, and pro-inflammatory cytokine release [9]. HIF1α seems to play a critical role in supporting glycolysis and polarization, even in normoxic conditions [10,11]. HIF-1α is activated downstream of two main pathways, mTORC1/Akt axis and TLR/NF-κB [9,10,11]. Following LPS or LPS and IFNγ stimulation during infection, macrophages showed high levels of HIF1α and, accordingly, HIF1α deficiency affects glycolytic activity in murine macrophages. Conversely, HIF1α activation is not impacted by the inhibition of glycolysis since HIF1α is an upstream modulator of the glycolytic pathway under mTORC1 control [73]. HIF1α presence supports glycolysis through the transcription of HIF1α target genes, including phosphoinositide-dependent kinase-1 (Pdk1), phosphoglycerate kinase 1 (Pgk1), Glut1, and pyruvate kinase M2 (Pkm2) [10]. In accordance, GLUT1 expression is significantly higher in M1 than M2, as recently demonstrated by the greater uptake by M1 macrophages of CDr17, a glucose scaffold combined with Cy5 fluorophore, capable of targeting GLUT1 [74]. 

NF-κB signaling also plays a key role in macrophage metabolic rewiring. LPS stimulation induces Glut1 gene via NF-kB activation, driving the inflammatory immune responses in macrophages. Again, the long non-coding RNA HOTAIR promotes IKBα degradation, thus activating NF-κB pathway and inflammation-induced metabolic reprogramming, regulating Glut1 expression and glucose uptake [75]. Notably, it was found in exosomes secreted by various cancer cells and tumors [76,77,78]. In M1 macrophages, the activation of glycolysis is a crucial event necessary for NLRP3 inflammasome induction and immune response. It was demonstrated that the mTORC1-dependent glycolysis in which hexokinase-1 (HK1) undergoes rapid increment is strictly associated with an equally rapid increment of glycolytic metabolites and NLRP3 activation [79]. In this context, after LPS stimulation, IL-10 deals with downregulating glycolysis via mTORC1 pathway inhibition, thus suppressing inflammatory response. Accordingly, IL-10-deficient macrophages suffer an enhanced pro-inflammatory activity correlated with abnormal glycolysis and mitochondrial damage [80].

In M1 macrophages, mTORC1 is also involved in promoting lipid synthesis via sterol regulatory element-binding transcription factor 1 (Srebp1) activation that sustains NADPH production in the PPP pathway [12]. 

Not only glucose but also glutamine is implicated in M1-like polarization [81]. Solute carrier family 15 member 4 (SLC15A4) drives metabolic homeostasis by affecting glutamine availability for the TCA cycle. In SLC15A4^−/−^ bone marrow-derived macrophages (BMMφ), mTORC1 activity decreased and AMPKα phosphorylation increased, resulting in increased glutamine use in the TCA cycle to cope with metabolic supply [13].

Conversely, M2 macrophage metabolism is mainly based on oxidative metabolism and FAO mediated by PPARγ to support tissue remodeling and repair [82,83]. The M2 phenotype and the relative metabolic shift are sustained by the IL-4-dependent activation of PI3K/Akt, where Akt mediates an increased consumption of glucose and also an expression of M2 genes, and the acute activation of mTORC1 blocks Akt in a negative feedback loop, fine-tuning the process [14]. In fact, IL-4/IL-4R signaling, amino acids levels, ADP/ATP ratio, and other metabolic parameters activate the PI3K/Akt axis that promotes H3 and H4 acetylation at promoters of M2 genes via ATP citrate synthase (Acly) activity and acetyl-CoA production [14]. Conversely, constitutively active mTORC1 impairs M2 metabolism, as demonstrated in a TSCΔ/Δ bone marrow-derived macrophages (BMDMs) model [84]. 

As a downstream effector of PI3k/Akt signaling, FoxO1 directs polarization toward M2 phenotype in the absence of metabolic stress. In fact, both in vivo macrophages and in vitro monocyte-derived macrophages (MDMs) exposed to hyperglycemic conditions re-directed their phenotype to M1 via a reduction in FoxO1 gene expression and its binding to the IL-10 promotor site [85,86]. 

mTORC2 is also involved in sustaining glucose utilization and OXPHOS. In fact, a loss of mTORC2 function due to Rictor deletion, associated with the lack of AKTs473 phosphorylation, compromises glycolysis and OXPHOS. Moreover, a lack of Rictor in murine macrophages leads to a lower differentiation toward the M2 phenotype, suggesting a crucial role of mTORC2/IRF4 in metabolic reprogramming and M2 macrophage activation [87]. 

The oxidative metabolism of M2 macrophages involves glutaminolysis. The anti-inflammatory metabolite α-ketoglutarate (αKG) promotes M2 activation via the Jumonji domain-containing protein-3 (Jmjd3)-dependent metabolic and epigenetic reprogramming, and suppresses M1-mediated proinflammatory responses by downregulating NF-kB pathway via prolyl hydroxylase domain (PHD)-dependent proline hydroxylation on IKKβ [88]. 

Dampening macrophage inflammation requires AMPK regulation of metabolism [89]. AMPK/SIRT1 signaling is involved in M2 macrophage polarization process and is responsible for activating anti-inflammatory transcription factors [90]. In fact, the pharmacological inhibition of either AMPK or SIRT1 caused a failure of polarization toward M2 [91]. Indeed, SIRT1-deficient macrophages are unable to inhibit acetylation of the pro-inflammatory mediator NF-κB/p65 and the upregulation of IKBα in the cytosol [91]. Moreover, AMPK sustains an anti-inflammatory phenotype to avoid insulin resistance. Hence, the development of insulin resistance in adipose tissue and the liver is exacerbated by the increase in inflammation in obese AMPK β1 bone marrow-null mice, thus causing severe clinical outcomes [31]. AMPK β1 is active in macrophages and phosphorylate acetyl-coA carboxylase (ACC) to regulate lipid metabolism, mainly during adipose tissue macrophage inflammation caused by saturated fatty acids. In fact, AMPK β1^–/–^ macrophages showed an enhanced inflammatory phenotype associated with lower levels of mitochondria and an impairment of FAO [31]. Specifically, in macrophages, AMPK is involved in mitophagy to control energy metabolism; impaired mitophagy correlates with the pro-inflammatory build-up of lipids, which, when in excess, cannot be fully oxidized, causing lipotoxicity, the production of mitochondrial ROS, and the activation of the NLRP3 inflammasome [32].

To further understand which metabolic pathway mainly contributes to the macrophage polarization process, the CoMBI-T dataset was developed as an integration model of mass spectrometry-based metabolic profiling and RNA-seq-based transcriptional profiling. In addition to recognizing the already-depicted metabolic features, glutamine metabolism and the UDP-GlcNAc pathway were identified as the required pathways to activate M2 program. In M1 macrophages, a metabolic break at isocitrate dehydrogenase (Idh) during the TCA cycle and the involvement of aspartate–amino succinate shunt to sustain carbon metabolism during nitric oxide production was identified as a key event [92].

Thus, pro-inflammatory M1 macrophages, responsible for mediating inflammatory responses, rely on glycolysis and PPP to meet their energetic requirements, whereas OXPHOS as well as FAO are downregulated. Conversely, the metabolic activity of M2 macrophages, which drives the resolution of inflammation, is mainly sustained by enhanced FAO and OXPHOS.

### 3.2. B-Cell Immunometabolism

B-lymphocytes are responsible for humoral immune response and can be classified in two main subpopulations named B1 and B2 [93]. B1 cells can be further subdivided in B1a and B1b cells and are long-lived self-renewing cells. They are mainly found in the peritoneal and pleural cavities and produce natural antibodies as part of innate-like immunity [94,95]. Instead, B2 cells, which represent the majority of all B cells, are short-lived and are metabolically quiescent recirculating cells that can be activated in secondary lymphoid organs to generate specific antibodies. Hence, they are essentially involved in adaptive immunity, can differentiate into memory cells, and undergo isotype switching [96]. 

As expected, it was observed that the metabolic profile of B cells is strongly correlated with both the development stage and activation state. Among metabolic pathways, mTORC and c-Myc signaling seem to be crucial for B-cell development due to their key role in regulating anabolic processes and upregulating glycolysis and OXPHOS. It was demonstrated that early B lymphopoiesis driven by interleukin-7 (IL-7) is mTORC1/Myc-mediated, and that mTORC1, but not mTORC2, is essential for pro-B to pre-B cell transition [15]. Interestingly, it was also observed that PTEN-mediated PI3K suppression is fundamental for IL-7 expression in pro-B cells, thus unpairing classical PI3K/mTOR signaling [15]. Moreover, a reduction in mTOR signaling impairs proliferation, cell growth, antibody production, and cell survival [97,98,99,100]. However, although mTOR signaling appears to play a key role in B-cell development, a sustained and uncontrolled activation of mTOR pathway blocks pre-B cell stage progression, inducing excessive cell growth, and enhances sensitivity to apoptosis following metabolic stress [101]. Specifically, Park et al. observed a complete block of development at the pre-B cell stage after deletion of the AMPK adaptor protein folliculin-interacting protein 1 (Fnip1) that caused a defective inhibition of mTORC1 complex by AMPK, thus resulting in a catabolic/anabolic imbalance [101]. 

As mentioned previously, the metabolic profile is not only dependent on the development stage, but is also cell-type specific. Clarke et al. demonstrated that B1 cells show higher rates of glycolysis and OXPHOS than B2 cells and observed an elevated expression of c-Myc [26], confirming the previous findings of Hayakawa et al. [102]. Moreover, compared with B2 cells, the B1 population is not only characterized by a higher expression of glucose transporter gene Glut1 and glycolytic gene Hk2, but also an upregulation of genes involved in fatty acid metabolism, such as acetyl-CoA carboxylase 2 (Acacb) and perilipin-3 (Plin3) [26]. 

The development and survival of B-cells are also influenced by the autophagic flux that plays different roles in both context- and cell-type-dependent manners. Autophagy seems to be dispensable for B-cell development since the deletion of autophagic gene ATG5 in mice at the outset of the pro-B cell stage (Atg5^f/−^ Mb1 cre) does not prevent pro-B-to-pre-B B2 cell transition [103]. However, other authors previously found a defective transition to pre-B cells in mice with ATG5^−/−^ fetal liver progenitors [104], but it is possible that the blockade of autophagic flux at this stage of embryonic life could affect hematopoietic stem cell maintenance [105], thus compromising lymphopoiesis from the earliest stages. As for B2 cells, also in the case of B1 cell development, autophagy process is not necessary [103]. However, autophagy seems to be essential for the survival of peripheral B cells, especially of peritoneal B1 cells [26,103]. Specifically, it was observed that autophagy is crucial for the self-renewal of B1 cell population by regulating exogenous fatty acid uptake and maintaining B1 lipid metabolism [26]. Moreover, unlike B2 cells, autophagy-deficient B1 cells failed to compensate for the metabolic stress with an increase in glycolytic flux [26], thus reflecting the unique immunometabolic profile of B1 lymphocytes. Autophagy blockade also affects B2-cell survival in peripheral lymphoid organs, albeit to a lesser extent, since a reduction in survival in a mouse model with ATG5 deletion in mature B cells (Atg5^f/−^ CD21 cre) was observed [103]. 

B-cell metabolic phenotype is also influenced by cellular localization. In germinal centers (GCs), for example, positively selected B cells in the light zone (LZ) show an activation of mTORC1 signaling required to support the subsequent clonal expansion in the dark zone (DZ) [106]. Moreover, mTORC1 signaling appears to be dynamically regulated since constitutive mTORC1 activation impairs affinity maturation and leads to a loss of competitiveness [106]. A significative role in mTOR signaling modulation could be played by hypoxia since it was observed that GC light zones are hypoxic [107]. In this regard, Cho et al. showed that the stabilization of HIF-1 in hypoxic conditions reduced mTORC1 signaling in B cells, thus limiting proliferation, isotype switching, and high-affinity antibody production [107]. Although hypoxia appears to be detrimental for B cells, it could regulate mTOR signaling output and generate a more stringent microenvironment for cell selection and survival. 

Along with physical environmental conditions, activation signals are also essential for B-cell metabolic switching. Naïve B cells, in fact, are maintained in a state of metabolic quiescence characterized by the activation of AMPK and the reduction in mTOR signaling [16]. The activation of B-cell receptor (BCR) promotes glucose uptake and glycolytic flux via the PI3K pathway [108], and since these steps take place in LZ [109], it is not surprising that this region is characterized by a strong PI3K activity [110]. On the other hand, GC B cells in DZ express FOXO1 transcription factor, which seems to be necessary for the DZ gene expression profile [110,111]. The activation state of B cells is crucial in controlling cell survival through metabolism regulation. It was observed that BCR stimulation alone is not sufficient for long-term survival since cells progressively lose glycolytic capacity and mitochondrial activity in the absence of a second signal [112]. Akkaya et al. showed how a second signal provided by helper T cells or via Toll-like receptor 9 (TLR9) is able to restore mitochondrial function [112]. B-cell activation is also associated with the upregulation of NF-κB signaling and c-Myc induction [113,114]. c-Myc expression enhances glycolytic flux [113] and could be directly related to NF-κB activation [115] via PI3K activity [116]. Moreover, NF-κB alternative pathway can boost B-cell metabolism upon B-cell activating factor (BAFF) receptor stimulation and subsequent TNFR-associated factor 3 (TRAF3) degradation. In fact, it was observed that TRAF3 deficiency results in an increased glucose uptake, glycolysis, and oxygen consumption in B cells [117]. Again, CD40 stimulation leads to an increased activity of NF-κB subunit c-Rel, which is able to reprogram GC-cell metabolism by regulating the expression of several glycolytic enzymes, such as phosphofructokinase (PFKM) and phosphoglycerate dehydrogenase (PHGDH), and several enzymes involved in FAO [27]. c-Rel also drives the expression of solute carrier family 7 member 6 (SLC7A6), which regulates the uptake of glutamine and other cationic amino acids required for anabolic reactions [27]. Furthermore, B-cell activation is also associated with HIF-1 stabilization [118]. Interestingly, although hypoxia represents the major mechanism for HIF-1 stabilization in LZ, the activation of B cells also allows for HIF-1 stabilization in normoxic DZ, where it promotes aerobic glycolysis and supports the rapid proliferation of centroblasts [118]. 

An important subset of B cells is represented by regulatory B cells (Bregs). They are found in both B1 and B2 lineages and produce IL-10, thus playing an essential immunosuppressive function in several physiological and pathological conditions [119,120]. Although the immunometabolic profile of Bregs is still unclear, they share several mechanisms with other B-cell subsets, since HIF-dependent glycolytic flux and mTOR activation are crucial for Bregs expansion and IL-10 production [17,18]. Importantly, as discussed in the following, Breg differentiation appears to be strictly mediated by environmental factors and conditions.

### 3.3. T-Cell Immunometabolism

T cells are a part of adaptative immunity, originating from bone marrow progenitors, and then migrating to the thymus for maturation [121]. They can be classified into three main subtypes: naïve T cells, effector cells, and memory cells. Naïve T cells are unassigned T cells and require antigen stimulation and external signals for maturation and survival. Based on the types of antigen-presenting cells, cytokines, and environmental conditions, naïve T cells are able to differentiate into T helper (Th or CD4^+^), cytotoxic T (Tc or CD8^+^), and regulatory T (Treg) effector cells [122,123,124,125]. 

Similarly to naïve B cells, naïve T cells are maintained in a metabolic quiescent state characterized by the basal uptake of nutrients and basal glycolytic flux and OXPHOS [126]. Recent thymic emigrant cells (RTEs), the earliest subset of naïve T cells, and naïve T cells in the periphery show a reduced mitochondrial mass, decreased levels of mitochondrial reactive oxygen species (mROS), and the downregulation of mTOR signaling [127]. Quiescence is also actively maintained by several mechanisms. Naïve T cells express high levels of PTEN [128], thus downregulating T-cell receptor (TCR) signaling [129] and suppressing PI3K pathway, whose activation is crucial for the development of T-cell effector phenotype [128,129]. The loss of PTEN, in fact, is associated with naïve T-cell proliferation and differentiation in the Th phenotype [129,130]. T-cell survival is mediated by interleukin 7 (IL-7)/interleukin 7 receptor (IL-7R) signaling, which regulates BCL-2 expression [28], and it was observed that stimulation must be intermittent rather than sustained in order to prevent cytokine-induced cell death (CICD) [131]. In addition, IL-7/IL-7R axis impairs cell atrophy in quiescent cells by improving glycolytic flux and sustaining basal metabolism and survival [28]. Interestingly, IL-7R expression in naïve T cells appears to be mediated by basal nuclear levels of NF-κB [132]. Quiescent cells are also characterized by the expression of Foxo1 transcription factor, which restrains cell metabolism by downregulating c-Myc and mTOR activity [19]. 

Conversely, activated CD4^+^ and CD8^+^ effector cells show a more dynamic metabolism and are reprogrammed to compensate for the higher nutrient requirements. Upon activation, for example, quiescent cells undergo a glycolytic switch, increase glucose uptake, perform aerobic glycolysis, and become more susceptible to glucose deprivation [133,134,135]. Notably, Cao et al. observed that CD8^+^ cells were more glycolytic than CD4^+^, owning to increased capabilities of growth and proliferation [66], suggesting that different effector cells may have slightly different metabolic profiles.

TCR and CD28 stimulations promote the activation of PDHK1 [20] and the expression of glucose transporter GLUT1 [21], respectively, thus sustaining aerobic glycolysis in effector cells. Accordingly, Macintyre et al. found that GLUT1 deficiency affects T-cell effector expansion and differentiation in vivo [22]. Quiescence exit and cell differentiation are strongly related to cell metabolic profile, which, in turn, is dependent on mTOR signaling [23,136]. mTOR can be activated through several mechanisms. Ray et al. demonstrated that interleukin-2 (IL-2)/interleukin 2 receptor (IL-2R) signaling promotes mTORC1 activation and Th1 differentiation via PI3K/Akt axis [137]. Moreover, the activation of TCR and IL-2 signaling are also able to increase amino acid uptake by upregulating the system L amino acid transporter SLC7A5, and thus activating mTORC1 complex [24]. Notably, SLC7A5 inhibition and deletion dampened cytokines production [29] and prevented mTOR-dependent glycolytic switch [24], respectively, and its expression appears to be mediated by NF-κB upon CD3/CD28 stimulation [29]. Upon stimulation, T cells also increase glutamine uptake through the expression of sodium-coupled neutral amino acid transporters SNAT1 and SNAT2, which are crucial for T-cell activation [138] and IL-2 and INF-γ production [139]. Similarly to B-cell metabolic reprogramming, T-cell activation and metabolic switch are associated with c-Myc activity, which enhances glucose catabolism [25]. mTOR signaling was also found to upregulate c-Myc post-transcriptionally, since a decrease in Myc protein level was observed, but not mRNA, in Rptor^−/−^ cells [23]. Conversely, Myc can upregulate mTOR signaling by increasing amino acid uptake. Wang et al. showed that the deletion of c-Myc in T cells impaired CD98 amino acids transporter transcription and mTOR pathway activation [25].

Another relevant mechanism in activated T cells involves the nuclear factor of activated T-cells (NFAT) that is induced by Ca2^+^ influx and regulates immune response and T-cell metabolism [140]. Notably, glycolytic metabolite phosphoenolpyruvate (PEP) is able to activate NFAT transcription factor by repressing sarco/ER Ca^2+^-ATPase (SERCA) activity, thus linking glucose availability with T-cell functions [141].

Autophagic flux also plays a relevant role in ensuring proper T cell survival, proliferation, and differentiation. In Atg5^−/−^ chimeric mice, Pua et al. found that Atg5^−/−^ CD8^+^ T lymphocytes displayed increased cell death in the periphery, and that Atg5^−/−^ CD4^+^ and CD8^+^ T proliferation was inefficient upon TCR stimulation [142]. Moreover, autophagy is induced during T-cell activation [143,144] and differently affects proliferation and differentiation depending on various T-cell subsets [145]. Autophagic flux is regulated by cytokines, which can both induce and inhibit autophagy. Several evidence suggests that anti-inflammatory cytokines exert an inhibitory effect, while pro-inflammatory cytokines induce autophagy [146]. Interestingly, the requirement of the autophagic negative regulator mTOR for T-cell activation and differentiation suggests the existence of other mechanisms of activation. In this regard, Botbol et al. found that autophagy activation is JAK-mediated in naïve and effector CD4^+^ T cells [143].

Among physical factors, hypoxia, and related HIF factors can influence T-cell metabolism. An engagement of TCR with CD28 results in the stabilization of HIF proteins regardless of oxygen tension [147], and this mechanism appears to be in part mediated by PI3K/mTOR activity [148]. Furthermore, cytokines such as IL-2 and IL-4 are able to stabilize HIF factors in normoxic conditions [147]. Interestingly, although HIF1α is not required for the metabolic switch after T-cell activation, it seems to play a role in sustaining and maintaining glycolytic flux during cell life [25]. Although the molecular mechanisms underneath the activity of HIF proteins are well described, the precise role of hypoxia in T-cell metabolism needs to be better understood. Moreover, T cells experienced different degrees of hypoxia in vivo and its effects could be beneficial or detrimental based on T-cell type and activation stage [149].

Unlike CD4^+^ and CD8^+^ T cells, Treg cells perform FAO and show an increased mitochondrial mass [150,151]. They are characterized by the expression of Foxp3 transcription factor, which drives OXPHOS [34] and is crucial for Treg immunosuppressive functions, including the inhibition of NF-κB and NFAT signaling in target T cells [30].

After antigen clearance, most of the effector cells die by apoptosis, but a small fraction of them survive and assume a memory cell-type phenotype. Notably, unlike CD4^+^ and CD8^+^ T effectors cells, memory cells are mainly supported by fatty acid oxidation (FAO), show a great mitochondrial respiratory capacity, and shift toward OXPHOS metabolism [152]. To support OXPHOS, memory cells perform mitochondrial fusion, while T effectors maintain mitochondrial fission tracts to promote aerobic glycolysis [153]. Moreover, memory cells utilize extracellular glucose for endogenous fatty acid synthesis, rather than using extracellular fatty acids to fuel FAO [154]. Interestingly, Bantug et al. found that mitochondria–endoplasmic–reticulum (ER) contact sites (MERCSs) represent crucial sites for coupling glycolysis and mitochondrial respiration and promoting a rapid recall response of memory CD8^+^ T cells via mTORC2/Akt signaling [155]. The minor dependence of memory cells on glycolytic flux and anabolic metabolism is further confirmed by the fact that AMPKα1-deficient cells are impaired in generating T memory cells [33]. Importantly, different subtypes of memory cells show different metabolic profiles. Ecker et al. observed that, under nutrient stress conditions, effector memory cells have limited capabilities to upregulate fatty acids synthesis compared to central memory cells, maintaining a sustained glycolytic flux and IFN-γ production, thus showing a greater ability to adapt to stressful conditions without losing effector functions [156].

## 4. Immunometabolic Interplay in Cancer

**Figure 1:**
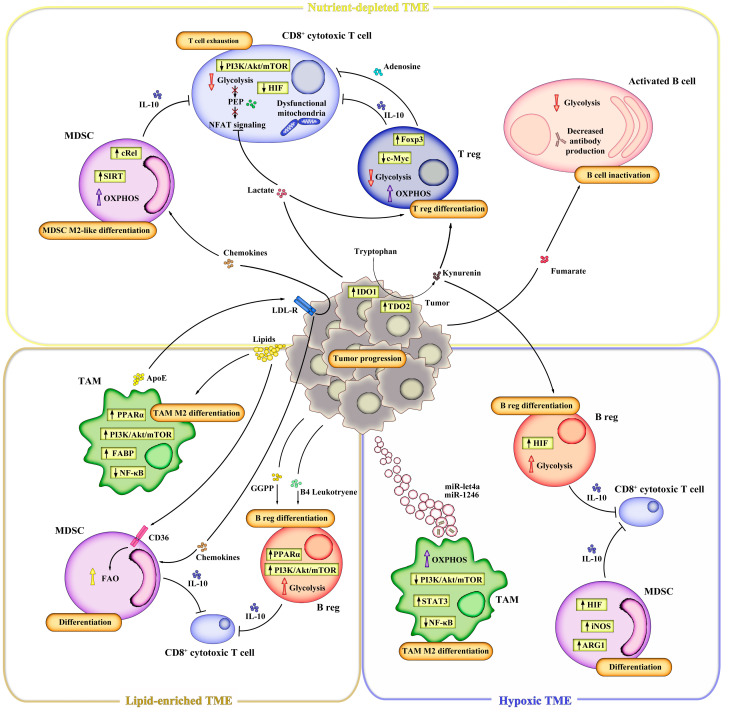
Schematic representation of metabolic immune reprogramming in immunosuppressive tumor microenvironment (TME). Nutrient depletion, lipid enrichment, hypoxia, and tumor-derived metabolites are the main factors driving immunosuppression and tumor progression by promoting (i) MDSCs recruitment; (ii) the differentiation of M2 macrophages, T and B regulatory cells; (iii) exhaustion of activated CD4^+^ and CD8^+^ T cells and inactivation of Ig-producing B cells. Directional arrows (up or down) indicate glycolysis (red), OXPHOS (violet), and FAO (yellow) rate.

Nutrient availability, physical conditions, and bioactive molecule balance contribute to creating an immunosuppressive TME. Many cues from TME can induce differentiation of Breg cells, Treg cells, myeloid-derived suppressor cells (MDSCs), and tumor-associated macrophages (TAMs) that are associated with reduced immunosurveillance and tumor progression [157,158,159] (Figure 1). CD4^+^ and CD8^+^ T effector cells are essential for tumor immunosurveillance [160]. However, in tumors, the immunosuppressive microenvironment impairs T-cell functions by reprogramming their metabolism. Importantly, several immunosuppressive mechanisms in T cells involve the modulation of cellular metabolism, such as the expression of the cytotoxic T-lymphocyte-associated antigen 4 (CTLA-4) and programmed death 1 (PD-1) immune checkpoints and their ligands B7-1/B7-2 and PD-L1/PD-L2. Although in healthy conditions, these factors orchestrate immune tolerance and prevent autoimmune disorders [161], in several diseases, including chronic viral infection and cancer, they may suppress T-cell functions excessively, thus impairing a proper immune response [162]. Upon receptor engagement, in fact, CTLA-4 and PD-1 block glycolytic flux, thus dampening T-effector-cell activity [163]. Inhibited T cells are unable to clear cancer cells and show a phenotype that is defined as “exhausted”. Notably, exhaustion is a dynamic process in which T cells experience several phases and metabolic changes characterized by a progressive loss of effector functions and the adoption of distinct metabolic profiles, ranging from “progenitors” to “terminally” exhausted T cells (Tex) [164,165]. Recently, many efforts have been made to rewire T-cell metabolism and restore T-effector activity, including CTLA-4 and PD-1 blockade that are able to rewire T-cell metabolism and partially restore T-cell functions. However, these approaches present several limitations [166]. A better understanding of the immunometabolic interplay in TME and the molecular mechanisms that lead to T cell exhaustion is needed to improve immunotherapy-based therapeutic strategies.

### 4.1. TAM Metabolism in Cancer

TAMs constitute the main component of the immune infiltration in tumors. The capacity of TAMs to condition tumor progression is strongly associated to specific metabolic reprogramming influenced by nutrients and oxygen availability in the TME and, cytokines, growth factors, as well as metabolic products secreted by cancer cells and/or other immune and stroma cells [167].

TAMs exhibit a high grade of heterogeneity and plasticity in response to functional and metabolic cues during tumor progression, playing dynamically M1-like and M2-like roles [167,168,169]. In this regard, macrophages (MΦs) co-cultured with tumor spheroid cells showed tumoricidal capacity compatible with M1 phenotype, but prolonged expositions induced MΦs to switch toward an M2 phenotype, supporting tumor development in a model of breast cancer [170].

A comparative proteomic analysis of tumor-extract-stimulated macrophages (TES-TAMs) revealed that glycolysis and inositol phosphate metabolism reprogramming sustain TAM differentiation [171].

The increased glycolytic activity in TAMs correlates to the promotion of tumor invasion and metastasis [172]. In fact, human peripheral blood monocytes co-cultured with pancreatic ductal adenocarcinoma (PDCA) cell lines face metabolic changes and upregulate glycolytic genes to support angiogenesis, extravasation, and EMT, while this metabolic rewiring does not occur in macrophages differentiated through co-culture with normal pancreatic cells [173]. According to Choi and colleagues, the gene expression profile of markers related to the glutamatergic signaling also changes in TAMs when they are exposed to glioblastoma cells and not to normal human astrocytes, showing a significant increase in the expression of GRIA2, SLC1A2, SLC1A3, and GLUL genes [174] and the activation of an immunosuppressive response.

TAM polarization toward M2-like pro-tumoral phenotype is also closely related to lipid metabolism that is, in turn, associated to PI3K/Akt pathway activation and NF-kB pathway shutdown. In a mouse model of gastric cancer, it was demonstrated that PI3K-γ induction in TAMs occurred through the accumulation of tumor-derived lipids or exogenous lipid supply [175]. In another study, ovarian cancer stem cells co-cultured with the murine macrophage cell line Raw264.7 favor the M2 polarization by increasing the relative protein level of PPARγ and decreasing NF-kB activation associated with pro-inflammatory M1 phenotype [35]. Moreover, the deletion of ABCG1 in macrophages derived from bladder carcinoma and melanoma caused free cholesterol intracellular accumulation and an increase in NF-kB p65 phosphorylation that promotes TAM re-polarization to an M1 phenotype, thus suggesting how, in this context, ABCG1 deficiency is associated with p65 activation to counteract tumor growth [176]. About cholesterol metabolism, apolipoprotein E (ApoE) produced by macrophages has been described as responsible for the immunosuppressive TME in PDAC. Mechanistically, macrophage-derived ApoE binds to LDL receptor and promotes NF-kB pathway activation and the subsequent release of CXCL1 and CXCL5 that, in turn, chemoattract immature myeloid cells to suppress CD8^+^ T-cell infiltration [177]. The relevance of lipid metabolism in TAMs is further sustained by the evidence identifying A-FABP, a member of the family of fatty acid binding proteins (FABPs), as a new functional marker for pro-tumoral TAMs involved in regulating IL-6 production through NF-kB/miR29b pathway [178].

Among pro-tumorigenic stimuli, hypoxia deeply affects TAM metabolism, although controversial findings have been reported that describe TAM as characterized by either a glycolytic phenotype or an oxidative one, likely due to different experimental settings, including duration and degree of hypoxia.

In hypoxic conditions, Quian et al. demonstrated that the enriched release of exosomes loaded with suppressive miR-1246 by glioma cells induced M2 polarization by activating STAT3 pathway and inhibiting NF-kB pathway [179]. Also, let-7a miRNA causes the polarization to M2 in TAMs and the metabolic shift to OXPHOS via the downregulation of insulin/Akt/mTOR pathway [180]. On the contrary, Arts and colleagues conducted a transcriptome analysis of TAMs co-cultured with thyroid cancer cells, showing the early activation of PI3K/Akt/mTOR pathway and HIF1α upregulation that sustained the glycolytic flux [181]. An additional study by Zhihua et al. further supported the glycolytic phenotype of TAMs under hypoxic conditions in human gastric cancers. Accordingly, the authors showed that the activation of the HIF-1α/miR-30c/REDD1/mTOR signaling pathway was associated with an increased glycolysis and the generation of an immunosuppressive TME [182]. Accordingly, the abnormal activation of REDD1, the negative regulator of mTORC1, hampers glycolysis, causing angiogenesis. Instead, REDD1-deficient hypoxic TAMs compete with tumor endothelial cells for glucose usage by upregulating Glut1 and thus preventing metastasization [183].

### 4.2. MDSC Metabolism in Cancer

Myeloid-derived suppressor cells (MDSCs) are a heterogeneous population of myeloid cells that often derive from altered myelopoiesis or from reprogrammed neutrophils and monocytes, which have been chronically exposed to inflammatory stimuli like cytokines and growth factors (GM-CSF, M-CSF, IL-6, IL-1β, etc.) often present in the TME [89]. Given that they are mainly associated with pathological conditions, their metabolic profile is described only in this section. MDSCs are divided into two groups according to the cell lineage of derivation: granulocytic/polymorphonuclear MDSCs (G-MDSCs or PMN-MDSCs) and monocytic MDSCs (M-MDSCs). MDSCs are deputed to create an immunosuppressive TME, where they rapidly expand, supported by metabolic changes [184].

During maturation, MDSCs are metabolically active, mainly relying on anaerobic glycolysis. Specifically, the interaction with cancer cells and the stimulation by GM-CSF drive MDSCs to a high utilization of glycolysis. In fact, M-MDSCs and G-MDSCs from tumor-bearing mice show high levels of glycolysis compared to the normal counterpart, and this metabolic choice prevents excessive ROS production and apoptosis. In this contest, the ATP produced by glycolytic MDSCs is progressively consumed, favoring the activation of catabolic reactions by the phosphorylation of AMPK that acts as an energy sensor. Notably, MSC-1-immortalized cells, derived from primary MDSCs, abolish their immunosuppressive phenotype and become unresponsive to AMPK in the presence of iNOS and ARG1 inhibition, suggesting the importance of the co-activation of AMPK, iNOS, and ARG1 to sustain the upregulation of central carbon metabolism that, in turn, sustains the MDSC immunosuppressive functions [185]. In tumor M-MDSCs with high glycolytic rates, several signaling pathways were downregulated, but the upregulation of phosphor-mTOR has been depicted as the main difference between tumor M-MDSCs and splenic M-MDSC. Therefore, Deng et al. speculated that targeting mTOR with rapamycin could be a way to exploit the activation of autophagy for reducing glycolysis and obtain a reduction in MDSCs’ immunosuppressive activity [89]. Recently, in NSCLC, the presence of a specific subpopulation of MDSCs, whose activation is supported by TGFβ released from NSCLC cells, has been documented for the first time. TGFβ promotes the mTOR-dependent HIF1α stabilization to induce CD39^+^ CD73^+^ subset of MDSCs involved in inhibiting the antitumor function of NK cells and effector T cells [186]. CD39^+^ CD73^+^ MDSCs have also been traced in ovarian cancer, where metformin treatment on MDSCs purified from peripheral blood of ovarian cancer patients or healthy donors showed promising results in restoring anti-tumor T-cell immunity by inhibiting HIF1α pathway [187]. Interestingly, it was shown that tumor MDSCs exposed to hypoxia upregulate the expression of inos and argI via HIF-1α, thus acquiring the ability to suppress antigen-nonspecific T-cell functions and focus on the immune suppression they drive in TME.

Although these findings pointed out a prominent role of glycolysis in sustaining MDSC immunosuppressive phenotype, this association is still debated. Indeed, some reports showed how an increased glycolytic flux was associated with a re-direction of MDSCs toward an M1-like TAM phenotype with reduced immunosuppressive functions, sustaining the hypothesis of a sharp mechanistic connection between immunosuppressive myeloid cells [188]. It seems that SIRT1 deficiency is associated with an increased mTOR/HIF1α signaling that regulates the metabolic reprogramming of MDSCs by activating glycolytic activity and differentiation toward the M1-like phenotype [189]. Also, Li and colleagues described a metabolic phenotype similar to M1, in Rel−/− MDSCs. Accordingly, c-Rel inhibitors reduced the suppressive properties of MDSCs through the reprogramming of their metabolism, by enhancing glycolysis in place of mitochondrial respiration. Moreover, Rel knockout MDSCs lack the expression of Cebpb and arginase 1 but Cebpb-overexpressing Rel–/– MDSCs recover immunosuppressive activity and OXPHOS flux [190]. 

A great bulk of evidence suggests the key role of lipid metabolism reprogramming in maintaining MDSC immunosuppressive functions in the TME. In vivo investigations in mice showed that high-fat diets increased MDSC differentiation from bone marrow precursors and boosted their suppressive phenotype, and obesity correlated with MDSC accumulation and a reduced CD8^+^ T cell/MDSC ratio in several tissues [191,192]. Lipids released from tumor cells are uptaken by MDSC via the upregulation of CD36 receptor and cause a metabolic switch from glycolysis to FAO in tumor-associated MDSCs. Accordingly, either CD36 ablation or FAO inhibition reduces tumor growth and improves response to both chemotherapy and immunotherapy [193,194].

### 4.3. B-Cell Metabolism in Cancer

Although little is known about the immunometabolism of B cells in cancer, their role in both immunosurveillance and tumor promotion is relatively well understood. To promote immunosurveillance, B cells can produce several cytokines [195], act as antigen-presenting cells to trigger anti-tumor T-cell response [196], and release antibodies, thus inducing antibody-dependent cellular cytotoxicity (ADCC) [197]. In the TME, B cells may be localized within tumor-associated tertiary lymphoid structures (TLSs) that comprise T cells, dendritic cells, and B-cell-rich areas containing germinal centers (GCs) [198]. Importantly, the localization and the composition of these aggregates are correlated with prognosis. In fact, a high degree of tumor-infiltrating B-cell TLSs correlate with a better outcome compared to peritumoral low-density TLSs [199,200]. Infiltrating B cells, however, are also able to produce a protumoral response. Indeed, B-cell-derived IL-10 was associated with a reduced T-cell-mediated antitumor response in vivo although this effect could be dependent on the tumor progression stage [201]. Maglioco et al. found that B-cell depletion prior to tumor inoculum promoted cancer growth, while B-cell depletion after tumor implantation inhibited tumor growth and delayed the onset of tolerance [202]. Other findings showed that the continuous production of antibodies in the TME can exert a protumoral effect through the formation of immune complexes and the subsequent promotion of invasion and inflammation [203,204]. Due to their seemingly conflicting roles in the immune response against tumor, a better understanding of the mechanisms regulating B-lymphocyte activity and its associated metabolism is undoubtedly needed. Nutrient competition in the TME certainly could play a key role in affecting B-cell antitumoral functions. A durable antibody response is mediated by long-lived plasma cells (LLPCs) that take up more glucose than short-lived plasma cells (SLPCs) [205]. Accordingly, low glucose availability affects glycolytic flux and inhibits B-cell differentiation into IgG-producing cells [206]. In a such glucose-depleted environment like the TME, B cells could use glutamine as an alternative carbon source [207], but also, this metabolite could become insufficient at some point, leading to the impairment of B cells’ functions.

Several findings showed that cancer-derived metabolites can affect B cells’ antitumor activities. An example is represented by kynurenine that is a tryptophan-derived catabolite produced by indoleamine-2, 3-dioxygenase (IDO1) and tryptophan-2, 3-dioxygenase (TDO2) [208]. These enzymes are frequently overexpressed in cancer cells and are associated with a poor prognosis [209,210]. By binding with aryl hydrocarbon receptor (AHR), kynurenine is able to regulate the expression of IL-10, promote the differentiation of B cells into immunosuppressive Bregs, and inhibit B-cell differentiation into a pro-inflammatory phenotype by downregulating cytokines, such as IL-2 and TNFα [211]. Another metabolite that affects B-cell function is fumarate; some tumors have mutations of the tricarboxylic acid cycle enzyme, fumarate hydratase (FH), that causes fumarate accumulation in the TME [212] Cheng et al. found that an excess of fumarate blocks the activation of B cells by covalently inhibiting tyrosine kinase LYN, thus interfering with BCR signaling [213]. Several inflammatory mediators such as leukotriene B4 are produced by enzymes often overexpressed in cancer cells [214], and their presence in TME contributes to tumor growth [215]. Breast cancer-derived leukotriene B4 activates the regulator of lipid metabolism PPARα in B cells, inducing their differentiation into immunosuppressive Bregs and promoting immunoevasion and tumor growth [216].

Recently, cholesterol metabolism and derived metabolites also seem to play relevant roles in immunosuppression [217]. In this regard, Bibby et al. found that the intermediate metabolite geranylgeranyl pyrophosphate (GGPP) is able to drive IL-10 production via the activation of PI3K/Akt pathway and the subsequent phosphorylation and suppression of GSK3β on Ser9 [218]. Metabolic reprogramming into immunosuppressive phenotype could also be mediated by hypoxia. Xianyi et al. showed that HIF-1α induced IL-10 expression and promoted glycolysis, thus facilitating CD1dhiCD5^+^ regulatory B-cell expansion [17].

### 4.4. T-Cell Metabolism in Cancer

Upon activation, effector T cells engage in aerobic glycolysis and show a high glucose demand to properly function [135]. Chang et al. showed that tumor-infiltrating lymphocytes (TILs) compete with cancer cells for glucose, and when this metabolite is scarce, a downregulation of mTOR activity, glycolytic flux, and interferon-γ IFN-γ production by TILs is observed [219]. Interestingly, aerobic glycolysis is not required for the survival and proliferation of T cells, but appears to be essential for cytokine production [134]. Moreover, metabolically reprogrammed TILs overexpressing phosphoenolpyruvate carboxykinase 1 (PCK1) increase levels of the glycolytic metabolite phosphoenolpyruvate (PEP) and upregulate the nuclear factor of activated T-cell (NFAT)-dependent IFN-γ production, thus enhancing their anti-tumoral activity [141]. Accordingly, the inhibition of glycolysis in tumor cells sustains T-cell antitumor activity, while, conversely, the overexpression of glycolysis-related genes in tumors impairs T-cell-mediated tumor regression [220]. Interestingly, although glycolytic flux is essential for T-cell effector functions, it was observed that the inhibition of glycolysis in CD 8^+^ cells promotes the formation of long-lived memory CD8^+^ T cells instead of short-lived effectors, thus improving the antitumor response [221] and highlighting the importance of properly regulating the glycolytic flux balance in order to obtain an efficient anticancer effect.

Due to their high rate of glycolysis, other than depleting TME from glucose, cancer cells secrete large amounts of lactate, which can impair T cells’ functions. An excess of lactic acid is able to block the function of monocarboxylate transporter-1 (MCT-1), leading to its accumulation in T cells, where it perturbs their metabolism and interferes with their functions [222]. Moreover, elevated levels of lactic acid also diminished NFAT transcription factor activity, with a subsequent decrement in IFN-γ production [223]. Importantly, if, on one hand, lactate interferes with T-cell-effector activity, on the other hand, it promotes Treg functions, thus contributing further to the establishment of an immunosuppressive TME. In Tregs, in fact, Foxp3 transcription factor impairs c-Myc expression, thus suppressing glycolysis, inducing OXPHOS, and enabling Treg to metabolize lactate, promoting their survival in low-glucose, lactate-rich environments [224,225]. Like glucose deprivation, amino acid availability and competition in TME can also affect T-cell functions. Blagih et al. observed that, in response to glucose limitation and subsequent AMPK signaling activation, glutamine was able to support effector T cells by supporting OXPHOS [226]. However, Leone et al. found that pharmacological glutamine blockade suppressed both glycolysis and OXPHOS in cancer cells but not in TILs, in which, in spite of a decreased glycolytic flux, they observed an upregulation of oxidative metabolism and the development of long-lived memory-type phenotype with efficient antitumor activity [227]. They also found an exceptional ability of T cells in utilizing acetate as fuel for the TCA cycle, supporting the crucial role of acetate in T-cell functions and memory-type phenotype development [228,229]. Notably, this correlation between decreased glycolytic signaling and T-cell memory development is in accordance with the previously mentioned work of Sukumar et al. [221].

As well as glucose metabolism, amino acid metabolism also produces several catabolites that can exert an immunosuppressive activity. As in the case of B cells, kynurenine is also able to dampen T-cell functions. On one hand, kynurenine is capable of inhibiting the proliferation of CD4^+^ and CD8^+^ cells in a concentration-dependent manner [230]; on the other, it is involved in the regulation of Treg-cell immunosuppressive functions and differentiation. Interestingly, Siska et al. found that kynurenine can enhance β-oxidation and deplete T cells from fatty acids necessary for proliferation, thus promoting their apoptosis. In support of these findings, oleate/palmitate supplementation rescued CD4^+^ cell proliferation [231]. Regarding the immunosuppressive effect of kynurenine, Mezrich et al. showed that kynurenine–AHR signaling is necessary for the generation of FoxP3^+^ Tregs [232]. Moreover, the expression of IDO in surrounding cells could lead to a depletion of tryptophan concentration in the nearby areas, thus inducing a general control nonderepressible 2 (GCN2)-mediated stress response in Treg cells [233]. Mechanistically, amino acid-sensitive GCN2 inhibits the activity of mTORC2 with the subsequent downregulation of Akt phosphorylation at Ser473. Due to the role of Akt in suppressing Foxo protein activity, the inactivation of Akt promotes the expression of Foxo3 [234], which is crucial in regulating Treg suppression function [235,236,237]. Other metabolites that affect T cells’ functions include γ-aminobutyric acid (GABA) and cholesterol. Zhang et al. showed that B-cell-derived GABA is able to inhibit T-cell proliferation and cytotoxicity [238]. Moreover, cholesterol in TME induces endoplasmic reticulum (ER) stress and the subsequent activation of X-box binding protein 1 (XBP1), thus upregulating PD-1 expression, dampening glycolysis, and promoting the exhaustion of T cells [239].

Functional Tregs also release high levels of adenosine in the TME which, in turn, other than promoting FoxP3 expression (thus stabilizing themselves in an autocrine manner [240,241]), exerts additional immunosuppressive effects through several mechanisms, including the downregulation of CD28 co-stimulatory receptor activity in CD4^+^ cells [242], and the reduction in mTORC1 signaling and effector functions in CD8^+^ [243].

Tumor progression is associated with a certain grade of necrosis, which releases extracellular content in tumor interstitial fluid (TIF), affecting the quality and concentration of several factors. It was observed, for example, that necrosis increases the concentration of potassium ions in TIF by suppressing Akt-mTOR signaling and NF-κB activation upon TRC engagement [244]. Moreover, elevated levels of potassium ions impair nutrient uptake, thus suppressing effector functions and promoting autophagy [245]. As mentioned previously, hypoxia also plays a role in regulating cell metabolism and T-cell functions in TME. Palazon et al. showed that HIF-1α promotes glycolysis and the infiltration of CD8^+^ in murine breast cancer models [246], although, previously, Hatfield et al. observed an increase in TILs in pulmonary tumors after respiratory hyperoxia in mice breathing 60% oxygen [247]. In accordance with Palazon et al., Gropper and collaborators showed that culturing CD8^+^ cells under hypoxia results in a higher production of granzyme B and in a more efficient anti-tumor activity in mice compared to normoxic T cells [248]. Conversely, Najjar et al. found a decreased infiltration and T-cell functions in melanoma tumors with oxidative, but not glycolytic, metabolism, characterized by marked hypoxic areas. Importantly, by inhibiting oxidative metabolism in tumor cells, they also observed a significative increase in T cells’ effector functions associated with reduced hypoxia and an increased sensitivity to PD-1 blockade therapy, showing how oxidative metabolism in tumors could be associated with T cell exhausted phenotype [249]. The divergence among these findings could be explained by the different experimental conditions, cell differentiation stage and O_2_ concentration during T-cell priming, and an experienced degree of hypoxia, which influences the magnitude of HIF proteins’ stabilization [149].

Oxygen is also necessary for the generation of reactive oxygen species (ROS), whose balance plays a crucial role in regulating T-cell functions. Although mROS are important and support metabolism during T-cell activation via NFAT signaling [250], high levels of ROS impair mTOR and NFAT activity, resulting in the downregulation of c-Myc, thus affecting glycolytic flux [251]. In human renal-cell carcinoma, a high production of ROS in TILs is associated with an exhausted phenotype characterized by mitochondrial dysregulation and metabolic dysfunction [252]. Depolarized mitochondria and an increased mitochondrial mass were also observed in TILs in melanoma mice models [253], further confirming the need to preserve mitochondrial function in order to prevent T-cell exhaustion. In ovarian cancer, a decrease in glucose uptake affects protein N-linked glycosylation and induces ER stress with the subsequent activation of XBP1, which, in turn, impairs glutamine influx under glucose deprivation and causes mitochondrial dysfunction [254]. It was observed that TILs showed mitochondrial dysfunction when infiltrating human and murine tumors due to a progressive loss of PPAR-γ coactivator 1α (PGC1α), which regulates mitochondrial biogenesis. Mechanistically, persistent antigen exposure in cancer promotes the chronic stimulation of Akt and the subsequent repression of Foxo activity, thus perturbing mitochondrial homeostasis [255]. Interestingly, if, on the one hand, sustained Akt signaling could lead to T-cell exhaustion, on the other, Akt activity, as mentioned previously, is also crucial for T-cell-effector functions. It was observed, in fact, that PD-1 and CTLA-4 immune checkpoint receptors frequently expressed on TILs [256,257], are able to downregulate glycolytic flux by inhibiting Akt through different and synergistic mechanisms [258]. Importantly, cancer cells often express PD-L1 and PD-L2 [259,260,261], thus promoting the engagement of PD-1 on immune cells and causing T-cell exhaustion. Moreover, it was observed that PD-L1 expression on cancer cells also drives Akt/mTOR signaling [219,262], thus boosting tumor glycolysis with the subsequent depletion of environmental glucose and a restriction of T-cell functions [219]. Notably, the activation of NF-κB in cancer cells directly induces PD-L1 gene transcription, highlighting its potential role in affecting T-cell metabolism indirectly [263]. However, although PD-1 blockade therapy is able to restore T-cell functions and induce an initial response in patients, it also promotes the expansion of FoxP3^+^ Treg cells’ pool, causing hyperprogressive disease (HPD) [264]. In addition, due to a high antigen concentration and persistent stimulation, reactivated Tex cells after PD-1 blockade often became re-exhausted [265] and showed a terminally differentiated phenotype, characterized by shortened telomeres and sensitivity to senescence or apoptosis [266]. In order to improve the efficacy of immunotherapy, Chamoto et al. showed that metabolic activators of AMPK, mTOR, and PGC1α synergize with PD-1 blockade in mouse models, inducing mitochondrial biogenesis, promoting FAO and OXPHOS, upregulating Bcl2, and generating long-lived effector memory cells, thus leading to tumor regression [267,268]. Moreover, Gu et al. revealed an important role of NIK in maintaining metabolic fitness. They found that NIK was able to prevent hexokinase 2 (HK2) autophagic degradation by stabilizing glucose-6-phosphate dehydrogenase (G6PD)-NADPH redox system, thus sustaining glycolytic flux in NF-κB-independent manner. In addition, the expression of NIK increased the resistance to PD-1 engagement and prevented CD8^+^ T-cell exhaustion, promoting antitumor immunity in vivo [269].

## 5. Conclusions

The metabolic regulation of immune cells is strictly regulated by several pathways, including AMPK, PI3K/Akt, and NF-κB signaling, and is affected by multiple factors, including cell-to-cell interactions, released cytokines, nutrient availability, and active biomolecules.

The evidence gathered reveals the great plasticity of immune-cell metabolism and highlights that the proper fulfillment of immune functions requires a fine-tuning between glycolytic and oxidative metabolism in a context-dependent manner.

Therefore, to improve cancer therapy, multiple factors must be taken into account, including the fact that, as in the case of immune checkpoint blockade monotherapy, a single chemotherapeutic agent could affect several cells in TME and produce opposite effects. Moreover, it is also necessary to accurately standardize experimental conditions since, as observed by Ma et al., in vitro T cells showed a different metabolic phenotype from in vivo cells [270]. In light of these findings, reprogramming and controlling immune cell metabolism is, therefore, a hard challenge, especially in such dynamic conditions dictated by the establishment of tumor-specific microenvironments, resulting from different metabolic phenotypes observed across human cancer cells. Accordingly, as shown by Reinfeld et al., nutrient partitioning in TME is not always the same and may result in different metabolic competition models [271]. Although the understanding of immunometabolic interplay in TME is still a work in progress, the bulk of evidence to date suggests that tackling metabolic vulnerabilities of immune cells could provide a great opportunity to overcome immunosuppression and improve cancer therapy.

## Figures and Tables

**Table 1:** Signaling pathways mediating metabolic programs in immune cell subsets.

Pathway	Status (on/off)	Cell Type	Mediators	Metabolic Programs	Refs.
**PI3K-Akt-mTOR**	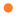	M1		Glycolysis	[9,10,11]
			SREBP1	Lipid synthesis NADPH production PPP pathway	[12]
			Slc15a4	Glutamine uptake	[13]
	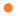	M2		Acetyl-CoA production and Acly activation	[14]
	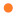	GCB	c-myc/IL-7	Glycolysis and OXPHOS	[15]
	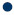	Naïve B	c-myc	Metabolic quiescence	[16]
	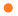	B reg		Glycolysis	[17,18]
	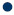	Naïve T	c-myc	Metabolic quiescence	[19]
	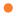	Activated/Effector T	c-myc; Glut1; PDHK1; SCL7A5	Glycolysis	[20,21,22,23,24,25]
**NF-κB**	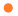	M1		Glucose uptake and glycolysis	[9,10,11]
	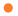	Activated B	c-myc	Glycolysis and OXPHOS	[26]
	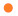	GCB	c-myc; Slc7a6	Glutamine uptake	[27]
	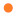	Naïve T	IL7-IL7R AXIS	Glycolysis	[28]
	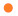	Activated/Effector T	SCL7A5	Amino acid uptake	[29]
	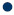	T reg			[30]
**AMPK**	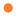	M2	ACC	Lipid metabolism	[31]
				Mitophagy	[32]
	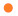	Naïve B		Metabolic quiescence	[16]
	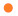	Memory T		OXPHOS and FAO	[33]
**Autophagy**	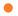	Activated B		FA uptake and lipid metabolism	[26]
**FOXP3**	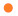	T reg		OXPHOS; FAO, increased mitochondrial mass	[34]
**PPARγ**	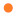	M2		Lipid metabolism	[35]

Abbreviations: GCB, Germinal center B cell; PPP, pentose phosphate pathway; Acly, ATP citrate lyase; B reg, B regulatory cell; T reg, T regulatory cell; OXPHOS, oxidative phosphorylation; FAO, fatty acid oxidation; FA, fatty acid; ACC, acetyl-CoA carboxylase.
